# Assessment of IL-18 values in septic acute lung injury/acute respiratory distress syndrome patients

**DOI:** 10.1186/cc9835

**Published:** 2011-03-11

**Authors:** T Masuda, Y Suzuki, T Shozushima, S Endo

**Affiliations:** 1 Iwate Medical University, Morioka, Japan

## Introduction

IL-18 is said to be involved in organ injury. We investigated the IL-18 values of septic acute lung injury (ALI) and acute respiratory distress syndrome (ARDS) patients.

## Methods

The subjects were 38 patients during the 3-year period from 2004 to 2007 from whom it was possible to collect a blood specimen within approximately 6 hours of the onset of septic ALI or ARDS. Their mean age was 67 years, and their mean APACHE II score was 29. Their SOFA score was 13, and their mean PaO_2_/FiO_2 _(P/F) ratio was 170. The P/F ratio was 246 in the ALI group and 135 in the ARDS group. There were four cases (10.5%) in the 28-day mortality group, and six cases (15.8%) in the 90-day mortality group.

## Results

The value of IL-18 in the died group was significantly higher than in the survived group (1,649 ± 1,056 pg/ml vs. 4,523 ± 2,798 pg/ml; *P *< 0.05), and in the ARDS group also significantly higher than in ALI group (2,467 ± 1,880 pg/ml vs. 1,314 ± 800 pg/ml); *P *< 0.05).

## Conclusions

These results suggested that IL-18 may play an major role in progression of ARDS in respiratory disorder as multiple organ failure.

